# Tannat grape pomace as an ingredient for potential functional biscuits: bioactive compound identification, *in vitro* bioactivity, food safety, and sensory evaluation

**DOI:** 10.3389/fnut.2023.1241105

**Published:** 2023-09-07

**Authors:** Victoria Olt, Jessica Báez, Romina Curbelo, Eduardo Boido, Miguel Amarillo, Adriana Gámbaro, Silvana Alborés, Natalia Gerez García, María Verónica Cesio, Horacio Heinzen, Eduardo Dellacassa, Adriana Maite Fernández-Fernández, Alejandra Medrano

**Affiliations:** ^1^Laboratorio de Bioactividad y Nanotecnología de Alimentos, Departamento de Ciencia y Tecnología de Alimentos, Facultad de Química, Universidad de la República, Montevideo, Uruguay; ^2^Graduate Program in Chemistry, Facultad de Química, Universidad de la República, Montevideo, Uruguay; ^3^Área Analítica Orgánica, Departamento de Química Orgánica, Facultad de Química, Universidad de la República, Montevideo, Uruguay; ^4^Área Enología y Biotecnología de la Fermentación, Departamento de Ciencia y Tecnología de Alimentos, Facultad de Química, Universidad de la República, Montevideo, Uruguay; ^5^Área Sensorial, Departamento de Ciencia y Tecnología de Alimentos, Facultad de Química, Universidad de la República, Montevideo, Uruguay; ^6^Departamento de Biociencias, Facultad de Química, Universidad de la República, Montevideo, Uruguay; ^7^Laboratorio de Farmacognosia y Productos Naturales, Departamento de Química Orgánica, Facultad de Química, Universidad de la República, Montevideo, Uruguay

**Keywords:** bioactive compound identification, food safety, functional biscuits, sensory analysis, Tannat grape pomace, wine byproduct, *in vitro* bioactivity

## Abstract

Grape pomace, the main by-product of wine process, shows high potential for the development of functional foods, being a natural source of bioactive compounds and dietary fiber. Thus, the present study proposes the development of five potential functional biscuits. The five formulations were achieved by varying the Tannat grape pomace powder (TGP, 10–20% w/w total wet dough) and sweetener sucralose (2–4% w/w total wet dough) content through a factorial design with central points. TGP microbiological and pesticides analysis were performed as a food safety requirement. Identification of bioactive compounds by HPLC-DAD-MS, *in vitro* bioactivity (total phenol content, antioxidant by ABTS and ORAC-FL, antidiabetic and antiobesity by inhibition of α-glucosidase and pancreatic lipase, respectively) and sensory properties of the biscuits were evaluated. TGP microbiological and pesticides showed values within food safety criteria. Sensory profiles of TGP biscuits were obtained, showing biscuits with 20% TGP good sensory quality (7.3, scale 1–9) in a cluster of 37 out of 101 consumers. TGP addition in biscuits had a significant (*p* < 0.05) effect on total phenolic content (0.893–1.858 mg GAE/g biscuit) and bioactive properties when compared to controls: 11.467–50.491 and 4.342–50.912 μmol TE/g biscuit for ABTS and ORAC-FL, respectively; inhibition of α-glucosidase and pancreatic lipase, IC_50_ 35.572–64.268 and 7.197–47.135 mg/mL, respectively. HPLC-DAD-MS results showed all the identified phenolic compounds in 20/4% biscuit (TGP/sucralose%) were degraded during baking. Malvidin-3-O-(6′-p-coumaroyl) glucoside, (+)-catechin, malvidin-3-O-glucoside, and (−)-epicatechin were the main phenolic compounds (in descendent order of content) found. The bioactive properties could be attributed to the remaining phenolic compounds in the biscuits. In conclusion, TGP biscuits seemed to be a promising functional food with potential for ameliorating oxidative stress, glucose and fatty acids levels with good sensory quality.

## 1. Introduction

Large amounts of agro-industrial by-products are generated each year because of agricultural crop processing. Despite their nutritional value, these by-products are often disregarded by industries, leading to their accumulation and becoming environmental pollutants (1). Among these industries, the wine industry stands out as a major contributor to agricultural waste. During wine elaboration, per 1,000 kg of grapes processed, ~200 kg of solid residues, mainly grape pomace composed of seeds and skin, are generated as a result. As a yearly outcome, it is estimated that 20 million tons of wine by-products are generated (2, 3). Therefore, it can be estimated that 6,000,000 kg of Tannat grape pomace were generated in 2022 (4). *Vitis vinifera* cv Tannat is a red grape variety that is currently cultivated in a few places in the world. It has become the emblematic wine variety of Uruguay, accounting for 1,575 hectares of the area of *V. vinifera* grapes planted in 2022 (4), representing 27% of the total wine grapes. Uruguay was the only country in the Americas where this grape was found 25 years ago. In recent years, several studies have been conducted in the country to better characterize its wine quality potential and to better understand the chemical composition of the grapes and wines (5, 6).

From the circular economy concept perspective, residues from wineries show high potential for new food products' formulation (2, 7). Wine and grape juice industrial residues are composed of high amounts of dietary fiber and phytochemicals, mainly phenolic compounds, including phenolic acids, flavonoids, and stilbenes (2, 8). Grape pomace polyphenolic content depends on the vinification method and contact time of the juice with skins, representing basic information for its application and profitability (9). These phenolic compounds have potentially beneficial effects on human health, being responsible for bioactive properties such as antioxidant, antiviral, antimicrobial, and anti-inflammatory activities depending on the grape variety (10). Specifically, Tannat grapes have a unique phenolic profile (5). Moreover, the hydro-alcoholic-acid extract of Tannat skin separated from grape pomace showed potential antioxidant, anti-inflammatory, antidiabetic, and antiobesity properties (11). Suitable strategies to manage and valorize the wine by-products both to reduce pollution and promote sustainable bioeconomy growth have emerged thanks to the circular economy concept (1). Among these strategies, grape pomace is being used as a substrate to cultivate oyster mushrooms as a way to promote the nutritional value of the spent substrate for ruminants (12). Moreover, due to the current trend toward natural and healthy food ingredients, grape pomace has gained recognition as an interesting ingredient that can serve as a substitute for synthetic antioxidants and enhance the nutritional value of the final product (13–15). The incorporation of grape pomace into food for the development of functional foods or its application as a nutritional supplement is being assessed for health promotion (8). Nowadays, as a consequence of consumers being more aware in their food consumption and health, industries and researchers are striving to develop innovative healthy food products keeping the product as natural as possible (16). The fortification of food products with functional ingredients, such as grape pomace, presents a promising approach to improve both consumer health and sensory properties (17). In this context, baked goods (muffins, breads, and brownies) have been prepared with wine grape pomace flour as a source of antioxidant dietary fiber with good consumers' acceptance (18). Among these baked products, biscuits are widely consumed in the world as a bakery snack; hence, they have emerged as excellent carriers for delivering essential nutrients and bioactive compounds such as polyphenols, vitamins, and pigments (17). However, to our knowledge, there are no studies on the identification of grape pomace phenolic compounds after baking to associate their presence to the bioactive properties of grape pomace food products.

Considering this context, the aim of this study was to develop different formulations of potential functional biscuits with the nutrition claims “source of fiber” and “no-added sugar” with Tannat grape pomace (TGP) incorporation, as a novel and sustainable ingredient for enhancing the nutritional composition, and explore their preventive effects against various chronic diseases. Thus, the biscuits were characterized in terms of their sensory and bioactive properties identifying the compounds that can potentially exert beneficial effects on consumers' health.

## 2. Materials and methods

### 2.1. Materials

Tannat (*Vitis vinifera* cv. Tannat) grape pomace (TGP) was provided by Bouza SA wine cellar (Montevideo, Uruguay). Chemicals for physicochemical assays such as dimethyl sulfoxide (DMSO), Folin reagent, gallic acid, 2,2′-azinobis-(3-ethylbenzothiazoline-6-sulfonic acid) diammonium salt (ABTS), fluorescein (FL) disodium salt, 2,2′-azo-bis(2-methylpropionamidine)dihydrochloride (AAPH), 6-hydroxy-2,5,7,8-tetramethylch-roman-2-acid (Trolox), α-glucosidase, 4-methylumbelliferyl-α-D-glucopyranoside (4-MUF), pancreatic lipase, 4-methylumbelliferyl-oleate (4-MUO), CH_3_COONa.3H_2_O, and buffer solutions Na_2_HPO_4_ and KH_2_PO_4_ were purchased from Sigma-Aldrich (St. Louis, MO, USA). All chemical reagents were of reagent grade. The ingredients to elaborate the biscuits (wheat flour, eggs, baking powder, sucralose, salt, and sunflower oil) were purchased from local stores in Montevideo (Uruguay).

For the GC-MS/MS, a Shimadzu GC-2010 Plus was equipped with an RTX-5 column of 30 m with a 0.25 μ layer, coupled to a TQ8050 MS/MS system, with the AART software for retention time adjustment (Shimadzu Co, Kyoto, Japan).

### 2.2. Tannat grape pomace (TGP) proximal analysis

Proximal analysis was carried out as described by AOAC (19) determining protein, fat, total dietary fiber, moisture, ashes, and total carbohydrates (by difference using protein, moisture, fat, and ash contents).

### 2.3. Sample preparation

#### 2.3.1. Tannat grape pomace powder

TGP was dried at 50°C in a conventional oven until constant weight (24 h). Dried TGP was subjected to size reduction using a domestic coffee grinder to achieve a particle size like that of commercial flour.

#### 2.3.2. Biscuit elaboration

Five formulations were prepared following a factorial design with central points to optimize % TGP and % sucralose according to the response variables: total phenol content, ABTS, and ORAC-FL. Two levels of substitution of wheat flour by TGP powder (10 and 20% w/w of total wet mass) and sucralose sweetener (2 and 4% w/w of total wet mass) were selected, as well as a central point with 15% of substitution by TGP powder and 3% of sucralose. In addition, control biscuits with different percentages of sucralose (2, 3, and 4% w/w in the total wet mass) and without substitution of TGP powder were formulated for comparison purposes. The formulations with the amounts of the ingredients are detailed in Table 1. The dough was prepared by mixing all the ingredients and then cut into equal shapes and sizes. They were baked in a conventional oven at 180°C for 12 min. The biscuits were subjected to size reduction obtaining a homogenous powder, packaged, and stored at freezer temperature (−20°C) for further bioactivity analysis.

**Table 1:** Amounts of the ingredients in the different formulations of biscuits.

**Biscuit formulations (TGP/sweetener%)**								
**Ingredient (%)**	**20/4%**	**20/2%**	**15/3%**	**10/4%**	**10/2%**	**4%**	**3%**	**2%**
Wheat flour	47.17	49.17	54.17	57.17	59.17	67.17	69.17	74.94
TGP powder	20	20	15	10	10	0	0	0
Baking powder	0.5	0.5	0.5	0.5	0.5	0.5	0.5	0.5
Salt	0.08	0.08	0.08	0.08	0.08	0.08	0.08	0.08
Sucralose	4	2	3	4	2	4	3	2
Eggs	14	14	14	14	14	14	14	14
Oil	14.25	14.25	14.25	14.25	14.25	14.25	14.25	14.25
Water	Qty Req	Qty Req	Qty Req	Qty Req	Qty Req	Qty Req	Qty Req	Qty Req

Qty Req: quantity of water required to form the dough.

Multiple regression analysis was performed fitting to the linear model with the following equation:


(1) $$\begin{array}{l} y=\;\beta_{0}+\;\beta_{1}X_{1}+\;\beta_{2}X_{2}+\;\beta_{12}X_{1}X_{2} \end{array}$$

where β_0_ indicates the intersection point, β_1_ and β_2_ the linear coefficients corresponding to % TGP and % sucralose, and β_12_ for the interaction between the independent variables (factors %TGP and % sucralose).

### 2.4. Food safety

Food safety analyses were carried out (microbiological and pesticide analyses) to determine TGP powder's suitability for human consumption as a food ingredient. Microbiological analysis was carried out following the limits established in the current regulations (20) and bibliographic data (21). The analyses were performed based on the methods described in the US Food and Drug Administration Bacteriological Analytical Manual (22, 23). The performed analyses were as follows: plate count of viable aerobic mesophiles, total coliforms, and fungi expressed in colony-forming units per gram of sample (cfu/g); Salmonella spp. search in a 25 g sample; and enterobacteria search in a 1 g sample.

Pesticide screening analysis of 107 residues in TGP was performed following an adaptation of the 21.01 official method and SANTE guidelines (24). In brief, 2 g of TGP powder was hydrated with 8 mL of deionized water and let stand for 30 min. Then, 10 mL of ACN was added and vortexed for 20 min at maximum speed. For the phase separation, 4 g of MgSO_4_ and 1 g of NaCl were added and agitated manually for 2 min and centrifuged at 3,000 *g* for 5 min. For the clean-up step, 125 mg of PSA and 100 mg of GCB were added to 4 mL of the ACN phase. The suspension was vortexed for 3 min and centrifuged for another 5 min. Two milliliters of the supernatant were taken, and the organic solvent evaporated under a gentle stream of N_2_ and the residue redissolved in 1 mL AcOEt solution was injected in the GC-MS/MS. Five replicas of the matrix extract were performed. Two were injected in the GC-MS/MS as such, and the other three were spiked with a mix of pesticide standards to check compound detectability at 0.01 mg/kg level. For pesticide identification, the criteria of the SANTE guidelines were followed: The retention time should not drift <0.1 min, and two transitions, the qualifier and the quantifier ions, for each parent were selected. The ions should completely overlap, having the same shape. The qualifier ion should have a S/N>3 whereas the quantifier a S/N>10. For screening purposes, only the qualifier was evaluated. As no pesticide was detected in the blank injections, a recovery experiment was performed: The mix of pesticides was added to 2 g TGP power in a crystallizer and mixed, and the solvent was allowed to evaporate overnight. The sample was then extracted as above.

### 2.5. Sensory evaluation

Sensory analysis performed by semi-trained judges and consumers was conducted in accordance with the Declaration of Helsinki. The study was reviewed and approved by the Ethics Committee for Research in Human Beings of the Faculty of Chemistry (Universidad de la República, Montevideo, Uruguay). Subjects gave written informed consent to participate in the study and were informed of the possibility of withdrawing from the study at any time.

#### 2.5.1. Sensory profile analysis

The five formulated biscuits were evaluated by 15 semi-trained judges (aged 30–60 years). The biscuits were presented to each judge at the same time and could be selected in the order the judge wished. They had to select the most appropriate attributes to describe them applying a rapid descriptive methodology (Flash Profile). Once the descriptive terms had been established, the five biscuits were evaluated using 10 cm long unstructured scales. The different attributes were anchored with “nil/not at all” and “high.” The evaluations were carried out in a sensory evaluation laboratory designed according to ISO 8589 (25).

#### 2.5.2. Consumer test

Consumer sensory analysis was carried out with the formulations (three biscuits) that showed significant differences in the sensory profile analysis by studying acceptability and perception. The three biscuits were presented to 101 consumers (aged 18–65 years) following a balanced complete block design. Consumers were recruited from the city of Montevideo (Uruguay) based on their interest in participating. For each biscuit, consumers indicate their overall liking using a 9-point hedonic scale. In addition, they had to answer check-all-that-apply (CATA) + just about right (JAR) questions (26) composed of 27 terms. Then, consumers responded to a brief survey of sociodemographic data.

### 2.6. Bioactivity assays

#### 2.6.1. Total phenol content

Total phenol content (TPC) was determined by the Folin–Ciocalteu method as described by Fernández-Fernández et al. (11). Measurement was performed after 30 min of incubation in the dark at 750 nm in a microplate reader (Thermo Scientific Multiskan FC model, Massachusetts, USA). The results were expressed as mg of gallic acid equivalents (GAE)/g of sample.

#### 2.6.2. Antioxidant capacity

Antioxidant capacity was determined by ABTS and ORAC-FL methods as described by Fernández-Fernández et al. (11). In both cases, the calibration curve was performed with Trolox (0.25–1.5 mM for ABTS and 0.01–0.08 mM for ORAC-FL), and the results were expressed as μmol of Trolox Equivalents (TE)/g of sample.

#### 2.6.3. Antidiabetic and antiobesity capacities

Inhibition of α-glucosidase and pancreatic lipase was assessed by measuring fluorescence (λexcitation = 360 nm, λemission = 460 nm) of the substrate (4-MUF-α-D-glucopyranoside) hydrolyzed by α-glucosidase and of the substrate (4-methylumbelliferyl-oleate) hydrolyzed by pancreatic lipase, respectively, for 30 min in a Varioskan Lux fluorimeter microplate reader (Thermo Scientific, Massachusetts, USA) (11). The results were expressed as the concentration of biscuit causing 50% inhibition (IC_50_, mg/mL) of α-glucosidase/pancreatic lipase.

### 2.7. Identification of Tannat grape pomace phenolic compounds

Bioactive compounds were extracted following the method described by Peña-Vázquez et al. (27) with some modifications (28). In brief, 50 mg of each sample was mixed with 1 mL of methanol/acidified water (0.1% formic acid) (80:20 v/v), followed by 90 min sonication, 1 min vortex, and centrifugation (10 min at 9,500 rpm), and the supernatant was collected. Afterwards, a re-extractaction of the residue was performed with 0.5 mL of methanol/acidified water (80:20 v/v), 25 min sonication, 1 min vortex and centrifugation (10 min at 9500 rpm). Extracts were mixed and stored at −20°C for further LC–MS analysis.

LC–MS analyses were carried out as described by Curbelo et al. (29) with some modifications (28). Phenolic compounds' separation was performed using an HPLC Kinetex C18-EVO reverse phase C18 column (5 μm particle size, 150 × 4.6 mm i.d., Phenomenex, California, USA) thermostated at 35°C. Mobile phase composition included: (A) 0.1% trifluoroacetic acid and (B) acetonitrile. The gradient was from 0 to 100% A for 3 min, from 4 to 30% B for 50 min, from 30 to 98% B for 5 min, and isocratic 98% B for 2 min, at a flow rate of 1.3 mL/min. Detection was performed at 280 nm. LC–MS analyses were assessed using a Shimadzu Triple Quadrupole MS detector (Shimadzu, Tokyo, Japan) using an electrospray ionization (ESI) interface (source voltage: 2.50 kV, capillary temperature: 250°C). Spectra were recorded in positive ion mode between m/z 100 and 2,000.

### 2.8. Statistical analysis

All experiments were performed in triplicate. Analyses were performed by analysis of variance (ANOVA), and significant differences were determined by the Tukey test (*p* < 0.05) using Infostat v. 2020 program. The results were expressed as means ± standard deviation (*n* = 3). For response surface graphs and multiple regression analysis, Statistica v. 7.0 and STATGRAPHICS Centurion 19 programs were used, respectively.

Sensory statistical analysis was performed by ANOVA (taking into account the judges, samples, and replicates) using XL-Stat 2021 software (Addinsoft, NY). For sensory profile analysis, the interactions assessor^*^biscuit and assessor^*^replicate were considered as variation factors, determining mean ratings and honest significant differences (Tukey test, *p* ≤ 0.05). Principal component analysis (PCA) of mean ratings was used to illustrate the relationship between variables and samples. For conducting consumers' sensory analysis, hierarchical cluster analysis was employed to identify consumer groups based on their overall liking of the products. The clusters were formed using Ward's aggregation criterion and the calculation of Euclidean distances between data points. To ascertain differences in the frequency distribution of gender, age, marital status, education, and consumption of non-nutritive sweeteners among clusters, the chi-square statistical test was used. Finally, an ANOVA was conducted on the overall liking data, considering sample, cluster, and their interaction as variation factors, determining mean ratings and honestly significant differences (Tukey test, *p* ≤ 0.05). Frequency of mention was obtained for the CATA questionnaire followed by performing the Cochran *Q*-test and Bonferroni test to identify significant differences in the selection of the terms. For each cluster, a correspondence factor analysis (CA) was performed on the contingency table obtained considering only significant terms according to the Cochran *Q*-test.

## 3. Results and discussion

### 3.1. Proximal analysis

TGP proximal analysis showed high dietary fiber content (Table 2) being beneficial for the development of food products with high nutritional quality. Dietary fiber content was higher than the one reported for Tannat grape skin (11) meaning the seeds contributed to fiber content. Compared to grape pomace from other grape varieties, TGP showed similar content to Manto Negro and higher content than Cabernet Sauvignon, Morio Muscat, Muller Thurgau, Merlot, and Pinot Noir (30). Regarding protein content, TGP showed similar values to Tannat grape skin, Manto Negro, Cabernet Sauvignon, Merlot, and Pinot Noir; surprisingly, fat content was higher than all these grape pomaces. TGP showed similar ash content to Cabernet Sauvignon, Merlot, and Pinot Noir (30). Altogether, the macro-component content shows the potential of TGP as an ingredient for the development of high-nutritional quality foods.

**Table 2:** Tannat grape pomace proximal analysis.

**Components**	g/100 g^*^
Proteins	13.13 ± 0.25
Lipids	8.97 ± 0.54
Total carbohydrates	70.63
Dietary fiber	63.82 ± 2.25
Ashes	7.27 ± 0.16

^*^Values on a dry matter basis.

### 3.2. Food safety

TGP microbiological analyses showed a viable aerobic mesophilic count of 1 × 10^3^ cfu/g, total coliform count lower than 3 cfu/g, absence of enterobacteria in 1 g, absence of *Salmonella* spp. in 25 g, and filamentous fungi count lower than 10 cfu/g. These results meet the food safety criteria stated by the Uruguayan regulations and bibliographic data (20, 21).

Regarding the analysis of pesticide residue of TGP, there was no detection of pesticides. There is no methodology available in the literature for TGP determination. The analytical methodology for sample preparation was based on the 21.01 methodology, the QuEChERS acetate-buffered method, with the addition of GCB in the clean-up step to eliminate the excess planar polyphenols (31). For pesticide residue determination, the suitability of the screening methodology assayed was checked, being possible to ensure the detection of the searched compounds. On the other hand, the usefulness of the sample preparation method was confirmed through recovery experiments at 0.01 mg/kg level, where the 107 pesticides searched for were detected. As no pesticide was detected, in the TGP sample, no further quantitative assays were performed.

Thus, TGP powder can be used as a food ingredient, making formulated biscuits an excellent food product for TGP bioactive compounds delivery for potentially enhancing consumers' health.

### 3.3. Sensory evaluation

#### 3.3.1. Sensory profile analysis

**Figure 1:**
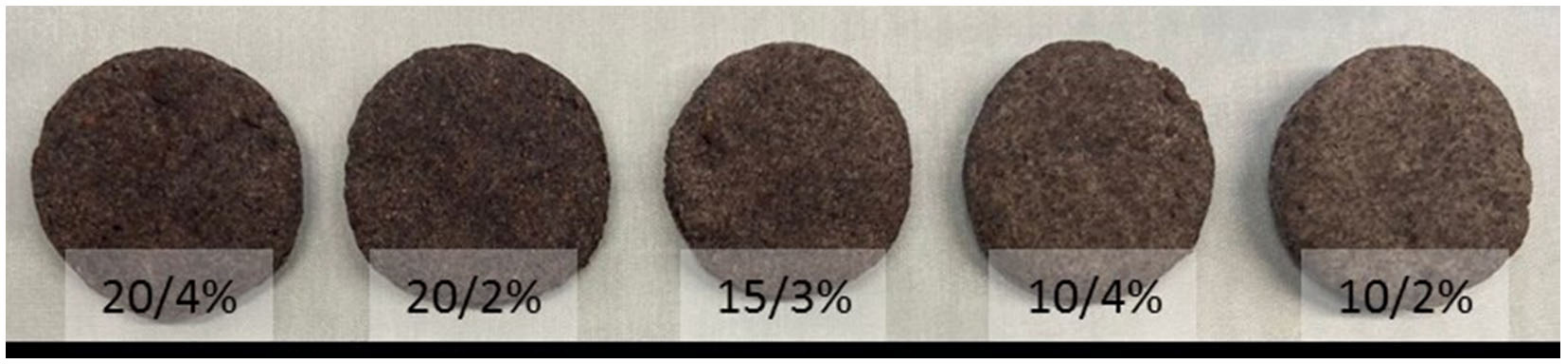
Biscuits evaluated by semi-trained judges.

The sensory profiles of the five biscuit formulations (Figure 1) are shown in Table 3. The biscuits with TGP powder increased significantly (*p* < 0.005) the intensity of all the sensory attributes evaluated, which could have an impact on the consumers' acceptability. The biscuits with 20% of TGP powder were described as harder, with a more intense color, with a greater grape flavor, more acid, with greater flavor intensity and persistence, and with a greater aftertaste than the biscuits with 10% of TGP powder. Some of these attributes have been used to describe the sensory profile of other grape pomace baked goods, showing increased acidity and hardness with the by-product addition (32). The biscuits with 4% of sweetener were perceived as significantly sweeter (*p* = 0.0066) than the samples with the same amount of TGP powder but with 2% sweetener. The biscuits with 15% of TGP powder showed an intermediate sensory profile.

**Table 3:** Mean values of biscuit intensities of the evaluated sensory attributes.

**Attributes**	**Biscuit formulations (TGP/sweetener%)**					**Significance level**
	**20/4%**	**20/2%**	**15/3%**	**10/4%**	**10/2%**	
Color intensity	7.3 a	7.1 a	5.5 b	3.2 c	2.8 c	< 0.0001
Hardness	6.0 a	5.7 a	3.9 b	2.0 c	1.8 c	< 0.0001
Crispness	6.4 a	5.4 a,b	4.4 b	2.2 c	2.0 c	< 0.0001
Dryness	5.7 a	4.8 a,b,c	5.2 a,b	3.2 c	3.6 b,c	0.0028
Sandness	5.7 a	5.0 a	4.6 a,b	3.8 a,b	2.6 b	0.0007
Sweetness	5.5 a	3.6 b	4.8 a,b	4.8 a	2.7 b	0.0066
Acid flavor	6.7 a	6.0 a	3.9 b	2.1 b,c	1.8 c	< 0.0001
Grape flavor	6.4 a	5.9 a,b	4.0 b,c	2.6 c	3.0 c	< 0.0001
Flavor intensity	6.5 a	5.6 a,b	4.2 b,c	3.0 c,d	2.4 d	< 0.0001
Aftertaste	3.9 a	2.9 a,b	2.6 a,b	2.2 a,b	1.6 b	0.0348
Flavor persistence	5.0 a	5.2 a,b	3.3 b,c	3.0 c	1.7 c	< 0.0001

Values within one row with different lowercase letters are significantly different according to the Tukey test (p < 0.05).

PCA was performed on the sensory data of the five evaluated biscuit formulations. The first two components (PC1 and PC2) accounted for 96.3% of the total variance (88.1 and 8.2% contributed by PC1 and PC2, respectively; data not shown). The color intensity, hardness, crispness, dryness, sandiness, sourness, grape flavor, flavor intensity, aftertaste, and flavor persistence contributed positively to PC1. These sensory descriptors were strongly correlated with each other. For PC2, sweetness contributed positively.

Three formulations of biscuits (20/4, 15/3, and 10/2%) showed significant differences in the sensory profile analysis by semi-trained judges, so they were selected for consumer analysis.

#### 3.3.2. Consumer test

The overall liking was 5.0, 5.3, and 5.2 for 20/4, 15/3, and 10/2% biscuits. These results were in agreement with those reported by Fernández-Fernández et al. (13) for Tannat grape skin biscuits and with data reported by Kuchtová et al. (33) attributing the decrease in overall liking to the astringent and bitter taste due to the increase in the amount of by-product. The identification of consumer groups was achieved by the hierarchical cluster analysis according to the overall linking toward the products. They were identified as clusters 1, 2, and 3, composed of 37, 27, and 37 consumers, respectively (Table 4), considering a minimum score of 6.0 on a 9-point hedonic scale as the lowest acceptable score for a product to be considered commercially viable (34). It is noteworthy that due to the low number of consumers in each cluster, the results may have limitations.

**Table 4:** Mean overall liking scores regarding cluster and biscuit formulation.

**Biscuit formulation**	**Cluster 1**	**Cluster 2**	**Cluster 3**	**Significance level**
	***n*** = **37**	***n*** = **27**	***n*** = **37**	
10/2%	6.5 b A	5.3 a B	3.7 b C	< 0.0001
15/3%	6.6 b A	4.2 b B	5.0 a B	< 0.0001
20/4%	7.3 a A	2.6 c C	5.6 a B	< 0.0001
Significance level	0.0091	< 0.0001	< 0.0001	

Lowercase letters in one column indicate significant differences according to the Tukey test (p ≤ 0.05). Capital letters in one row indicate significant differences according to the Tukey test (p ≤ 0.05).

Consumers of Cluster 1 showed significant differences (*p* = 0.0091) between the samples for the biscuit with the highest amount of TGP powder, the preferred one over the other two samples. The 20/4% biscuit, preferred by this group, was described as having adequate hardness, smooth texture, adequate sweetness, intense flavor, acid flavor, grape flavor, and being delicious. On the other hand, consumers of Cluster 2 preferred the biscuit with the least amount of TGP powder over the other two samples (*p* < 0.0001). The general liking decreased significantly with the addition of higher amounts of TGP powder showing quite low values of acceptability (below the commercial limit). The perception of these samples regarding negative attributes, such as not very sweet, dry, with aftertaste and strange taste, explain the low acceptability assigned by this group of consumers to all biscuits, indicating that Cluster 2 cannot be considered as potential consumers of TGP biscuits. Among the consumers of Cluster 3, significant differences (*p* < 0.0001) were also found between the evaluated samples, with the preference of the two biscuits with the highest amount of TGP powder over the sample 10/2%. This group assigned low acceptability scores to the biscuits evaluated, mainly to the ones that contained the least amount of TGP powder; therefore, they could be considered potential consumers of the biscuits with the highest % of TGP powder. The terms most used by consumers in Cluster 3 were adequate color, adequate hardness, slightly sweet, mild flavor, acid flavor, grape flavor, and strange flavor.

The attributes that were mentioned by the consumers and their frequency of mention for TGP biscuits were in agreement with Tannat grape skin biscuits (13), which were described as follows: from adequate color to too dark, of adequate crunch and being dry, adequate sweetness, with intense and persistent flavor, acid flavor, with adequate grape flavor.

### 3.4. Bioactivity assays

#### 3.4.1. Total phenol content (TPC) and antioxidant capacity

TPC and antioxidant capacity increased in the biscuits with the substitution of wheat flour for TGP powder compared with their control biscuits (Table 5). The biscuit with a higher percentage of TGP and sucralose (20/4%) showed the highest values of TPC and antioxidant capacity (*p* < 0.05). These results indicated that the phenolic compounds present in the TGP powder remained with antioxidant capacity in the biscuits after baking. These results are in agreement with those reported by Theagarajan et al. (35) where biscuits added with 6% grape pomace powder showed significantly higher total polyphenols and antioxidant capacity compared with biscuits with 4% of grape pomace powder and a control biscuit without the incorporation of the by-product. Similarly, the fortification of cakes with 4, 6, 8, and 10% of Grape Pomace Powder (GPP) resulted in a significant increase in the antioxidant capacity measured by DPPH and FRAP assays, compared to the control cakes with no addition of GPP. The same trend was observed by the authors, where the cake with 10% of GPP showed higher antioxidant capacity (36). Accordingly, the Tannat grape skin biscuit (20% w/w Tannat grape skin of total dough wet mass) showed increased antioxidant capacity compared to the control biscuit (*p* < 0.05) (13).

**Table 5:** Total phenol content (TPC) and antioxidant capacity were measured by ABTS and ORAC-FL of the different formulations of biscuits.

**Samples**		**TPC (mg GAE/g biscuit)**	**ABTS (μmol TE/g biscuit)**	**ORAC-FL (μmol TE/g biscuit)**
TGP biscuit	20/4%	1.858 ± 0.043^e^	50.491 ± 1.863^e^	50.912 ± 3.656^d^
	20/2%	1.575 ± 0.029^d^	43.808 ± 1.505^d^	37.111 ± 2.465^c^
	15/3%	1.242 ± 0.014^c^	31.888 ± 2.366^c^	33.332 ± 2.012^bc^
	10/4%	1.225 ± 0.119^c^	26.697 ± 1.364^b^	25.976 ± 2.211^b^
	10/2%	1.250 ± 0.023^c^	29.882 ± 2.057^c^	29.291 ± 5.718^b^
Control biscuit	4%	1.059 ± 0.048^b^	14.433 ± 1.099^a^	7.994 ± 1.147^a^
	3%	0.999 ± 0.023^b^	11.467 ± 2.101^a^	6.910 ± 1.841^a^
	2%	0.893 ± 0.048^a^	12.134 ± 0.514^a^	4.342 ± 1.790^a^

The results are expressed as mean values ± SD. Different letters indicate significant differences (Tukey, p < 0.05) between the mean values in the same analysis (per column).

**Figure 2:**
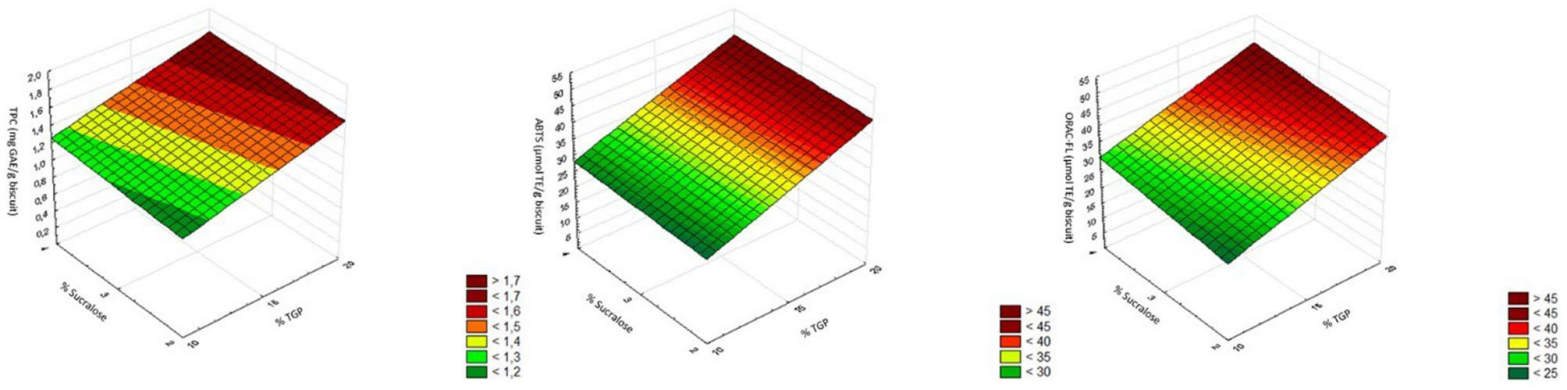
Response surface graphs of TPC, ABTS, and ORAC-FL determinations.

Figure 2 surface plots show the relationship between the TGP percentage and that of sucralose incorporated into the biscuits, presenting a linear association with the response variables. Multiple regression analysis fitted to Equation 1 resulted in the following three equations (Table 6): TPC = 1.38+0.24^*^TGP; ABTS = 35.02+9.43^*^TGP; ORAC-FL = 34.76+8.19^*^TGP. The non-significant coefficients (*p*-value > 0.05) were taken out from the analysis, showing that the response variables were only dependent on factor %TGP and positively influenced by this factor. The factor %sucralose was non-significant showing no significant influence on the response variables. *R*^2^ values indicated that the variability in TPC, ABTS, and ORAC-FL is poorly explained by the fitting of the model because of the low values (< 80%). The highest TPC and antioxidant capacity corresponded to the biscuit with the highest substitution of wheat flour by TGP powder (20%).

**Table 6:** Multiple regression analysis results for TPC, ABTS, and ORAC-FL response variables.

**Coefficients**	**TPC**	**ABTS**	**ORAC-FL**
Constant	1.38	35.02	34.76
%TGP	0.24	9.43	8.19
%sucralose	-	-	-
%TGP^*^%sucralose	-	-	-
*R*^2^ (%)	61.49	77.10	66.22
Adjusted *R*^2^	53.79	72.53	59.46
*p*-value	0.037	0.009	0.026

#### 3.4.2. Antidiabetic and antiobesity capacities

The use of enzyme inhibitors in the prevention and treatment of diseases is applied in diabetes to reduce postprandial glucose levels by the inhibition of carbohydrate-hydrolyzing enzymes such as α-glucosidase. In the same way, inhibition of lipases related to triacylglycerol absorption is used to reduce fat levels and thus treat obesity (37). Our results suggested greater inhibition of α-glucosidase and pancreatic lipase with the highest substitution of wheat flour by TGP powder (20%) in biscuits (Table 7), showing the potential of TGP powder as a functional ingredient for regulating hyperlipidemia and hyperglycemia. These inhibitory capacities may be attributed to grape phenolic compounds (37).

**Table 7:** α-glucosidase and pancreatic lipase inhibitory activity of the biscuit formulations.

**Samples**		**α-glucosidase inhibition**	**Lipase inhibition**
		**IC**_50_ **(mg/mL)**	**IC**_50_ **(mg/mL)**
TGP biscuits	20/4%	35.572 ± 0.549^a^	7.197 ± 0.811^a^
	20/2%	43.148 ± 5.223^ab^	8.573 ± 1.505^a^
	15/3%	55.415 ± 1.019^bc^	10.355 ± 1.129^a^
	10/4%	45.351 ± 6.963^ab^	37.434 ± 0.988^b^
	10/2%	64.268 ± 3.248^c^	47.135 ± 2.443^c^
Control biscuits	4%	58.528 ± 6.655^bc^	N I
	3%	69.257 ± 8.016^c^	N I
	2%	107.447 ± 13.827^d^	N I

The results are expressed as mean values ± SD. N I, No inhibition. Different letters indicate significant differences (Tukey, p < 0.05) between the mean values in the same analysis (per column).

When analyzing the TGP phenolic composition in flavan-3-ols, flavonols, and anthocyanins, as well as taking into account similar food matrices to TGP, an association of α-glucosidase and pancreatic lipase inhibition capacities can be made. Among anthocyanins, cyanidin and its glycosides have demonstrated α-glucosidase inhibition capacity (38). Proanthocyanidins have been reported for non-competitively inhibiting pancreatic lipase inducing its aggregation and subsequent stabilization of the aggregates (39). Regarding similar food matrices' phenolic composition to TGP, extracts of anthocyanin sources, which present flavonol glycosides (berry extracts, from black currant and rowanberry) such as TGP, have shown α-glucosidase inhibition capacity (40, 41). In addition, red fruit extracts from aronia (*Aronia melanocarpa*, A.), pomegranate (*Punica granatum L*., P.), and red grape (*Vitis vinifera*, RG) have shown high α-glucosidase inhibition capacity, being the anthocyanins, flavonols, and ellagitannins responsible for the inhibition (42). Particularly, the fractionation of the red grape extract showed higher inhibition capacity for the polymeric fraction (tannins such as condensed flavan-3-ols) when compared to anthocyanin (malvidin-3-glucoside, petunidin-3-glucoside, peonidin-3-glucoside, malvidin-3-acetylglucoside, coumaroyl derivatives of anthocyanins) and copigment fractions (hexoside of coumaric acid and isorhamnetin) (42). Other anthocyanin sources, blackthorn, açaí, and maqui blends, have been reported for inhibiting pancreatic lipase activity being negatively correlated with their anthocyanin content, as well as showing α-glucosidase inhibition capacity (43). Tannin-rich extracts from persimmon (*Diospyros kaki* Thunb.) have shown α-glucosidase inhibition capacity as well as glucose uptake and transport reduction in the Caco-2 cells model (44). A phenolic-concentrated extract of native Chilean red strawberry (*Fragaria chiloensis* ssp. *chiloensis f. patagonica*) has shown α-glucosidase (IC_50_ = 0.80 μg/mL) and pancreatic lipase (41.36% inhibition at 50 μg/mL) inhibition capacities (45). Phenolic extracts from Canadian lentil cultivars (*Lens culinaris*), mainly composed of flavonoids (kaempeferol glycosides, catechin/epicatechin glucosides and procyanidins) such as TGP, presented good α-glucosidase and pancreatic lipase inhibition capacities (46). The main responsible for these exerted capacities may be the flavonols (46).

On the other hand, berry seeds such as maqui seeds, which are composed of polyunsaturated fatty acids, tocols, tocotrienols, tocopherols, and sterols, have shown α-glucosidase (IC_50_ > 265.9 μg/mL) and pancreatic lipase (IC_50_ > 71.33 μg/mL) inhibition capacities (47). Fruit fractions of saskatoon berry showed similar IC_50_ values to the current work for α-glucosidase (IC_50_ > 23.60 mg/mL) and pancreatic lipase (IC_50_ > 81.90 mg/mL) inhibition capacities (48). In contrast, Merlot grape pomace extract presented no inhibition of α-glucosidase probably because of the low concentrations that were tested (< 250 μg/mL) (49). Particularly, Tannat grapes have shown α-glucosidase inhibition capacity (50), as well as Tannat grape skin (IC_50_ = 11.67 mg/mL) (13) and their extracts (lowest IC_50_ = 0.8886 mg/mL) (11). Moreover, a Tannat grape skin extract has shown pancreatic lipase inhibition capacity (IC_50_ = 2.431 mg/mL) (11). Extracts from red grape pomace, skin, and seeds presented α-glucosidase inhibition capacity (IC_50_ of 0.55, 0.30, and 0.36 mg/mL, respectively), as well as yogurt fortified with those extracts (51). In sum, TGP inhibition capacities can be attributed to grape phenolic compounds.

Regarding the inhibition capacities of foods with the addition of these phenolic compounds, the results of α-glucosidase inhibition are in agreement with the ones of a flatbread enriched with a source of oligomeric proanthocyanidins (white grape skin), showing increased activity compared to non-enriched food (52). The authors found that the inhibition increase was lower than expected (based on the grape skin added amount), probably because of proanthocyanin/protein complexes' formation with food matrix components which lowered solubility, hence reducing their accessibility for bioactivity determinations. Moreover, the baking temperature might have caused changes in those complexes as well as partial proanthocyanidins degradation (52). These statements are in agreement with the current results. Phenolic compounds can interact with food matrix macro-components such as lipids, proteins, and carbohydrates, changing their structure with subsequent changes in their bioactivity (53) by impairing their interaction with these enzymes.

In another work, blackcurrant pomace (by-product from fruit juice production) showed inhibition of glucose release during *in vitro* digestion when added to muffin formulations, by inhibiting starch digestive enzymes, counteracting pre-gelatinized starch (extruded wheat flour) hyperglycemic effect (54). Moreover, rye bread fortification with powders from Saskatoon berry fruits led to an increased inhibitory activity against α-glucosidase and pancreatic lipase (55). Kefir fortified with UAE-derived Sangiovese skin extract (from red grape pomace) presented α-glucosidase and pancreatic lipase inhibition (56). Grape seed powder inhibited cream (containing milk fat) lipolysis, whereas no inhibition was observed for beef fat, indicating that lipolysis may be determined by the dietary fat source of a meal (57). As to biscuits added with other food matrixes, a coffee fiber-containing biscuit showed a reduction of α-glucosidase activity (58). Biscuits with orange pomace have shown lower IC_50_ values (< 25 mg/mL, higher α-glucosidase inhibition capacity) (59) than TGP biscuits. In contrast, particularly biscuits with 20% of Tannat grape skin have shown higher IC_50_ values (>55 mg/mL, lower α-glucosidase inhibition capacity) (13) than 20% TGP biscuits.

Although red grape by-products have shown these capacities (11, 13, 60), to our knowledge, it is the first time that they have been determined in biscuits with grape pomace.

The biscuit 20/4% was the one that presented the highest antioxidant, antidiabetic, and antiobesity properties; therefore, LC-MS analysis was performed on this formulation.

### 3.5. Identification of Tannat grape pomace phenolic compounds

Regarding TGP phenolic profile (Table 8), flavan-3-ol, flavonol, and anthocyanin profiles agree with those of the grape not submitted to the vinification process (5). Particularly, the anthocyanin profile and part of the flavonol profile agree with the one from Tannat grape skin from grape pomace (13) and Tannat grape pomace (61), showing the characteristic p-coumaroyl derivatives of Tannat grape.

**Table 8:** Data on the identification of phenolic compounds composing TGP and TGP biscuit.

	**Compound**		**TGP**		**TGP biscuit (20% w/w)**	
			**Rt (min)**	**Area**	**Rt (min)**	**Area**
Chromatogram 280 nm	Phenolic acids	Cis-caftaric acid	6.496	34,450		
		Trans-caftaric acid	6.893	90,891		
		Protocatechuic acid	8.730	225,597		
		Trans-coutaric acid	12.313	265,757		
		p-coumaroyl hexose	12.457	573,977		
	Flavan-3-ols	Procyanidin trimer C2	4.628	39,983	4.376	974
		Procyanidin dimer B1	7.453	181,179	7.040	3,393
		Procyanidin dimer B3	8.022	303,632	7.562	26,005
		(+)-catechin	8.306	436,692	7.905	53,780
		Procyanidin trimer	9.104	199,322	8.852	7,084
		Procyanidin trimer	10.608	1,127,900	10.315	6,897
		Procyanidin dimer B4	11.459	328,747	10.862	2,461
		Procyanidin dimer B6	11.832	204,148	11.617	15,877
		(-)-epicatechin	12.457	839,734	11.835	32,999
		Procyanidin dimer galloylated	15.757	107,028	14.862	1,430
		Procyanidin trimer	16.024	89,969		
		Procyanidin trimer	16.324	341,893		
		Procyanidin dimer B2	17.158	161,716		
		Procyanidin dimer galloylated	17.579	302,107		
		Procyanidin dimer B7	22.827	200,876		
	Flavonols	Myricetin-3-O-galactoside	19.665	79,965		
		Myricetin-3-O-glucoside	21.814	31,333		
		Quercetin-3-O-galactoside	22.689	65,749		
		Quercetin-3-O-glucoside	23.466	162,693	23.165	17,380
		Siringetin-3-O-glucoside	26.790	71,502		
		Quercetin-7-O-neohesperidoside	28.236	160,861	28.013	4,425
		Quercetin aglycone	35.226	57,127	34.083	9,259
Chromatogram 520 nm	Anthocyanins	Delphinidin-3-O-glucoside			14.024	2,095
		Petunidin-3-O-glucoside	17.573	52,866	17.493	9,506
		Peonidin-3-O-glucoside	19.744	74,672	19.478	2,392
		Malvidin-3-O-glucoside	20.804	882,877	20.724	42,255
		Petunidin-3-O-(6′-acetyl)glucoside	26.547	34,547	26.743	1,275
		Peonidin-3-O-(6′-acetyl)glucoside	28.848	13,166		
		Malvidin-3-O-(6′-acetyl)glucoside	29.545	211,121	29.777	4,879
		Delphinidin-3-O-(6′-p-coumaroyl)glucoside	30.385	72,350	30.696	4,851
		Malvidin-3-O-(6′-caffeoyl)glucoside	32.285	192,783	32.599	3,931
		Cyanidin-3-O-(6′-p-coumaroyl)glucoside	32.677	28,817	32.893	1,373
		Petunidin-3-O-(6′-p-coumaroyl)glucoside	33.362	308,975	33.699	9,771
		Peonidin-3-O-(6′-p-coumaroyl)glucoside	35.785	195,123	36.016	5,279
		Malvidin-3-O-(6′-p-coumaroyl)glucoside	36.079	2,268,418	36.458	67,424

Rt, retention time.

LC-MS results (Table 8) showed that the main phenolic compounds present in TGP biscuit were malvidin-3-O-(6′-p-coumaroyl)glucoside, (+)-catechin, malvidin-3-O-glucoside, (-)-epicatechin, procyanidin dimer B3, and quercetin-3-O-glucoside, in descendent order, in contrast with TGP that showed malvidin-3-O-(6′-p-coumaroyl)glucoside, procyanidin trimer, malvidin-3-O-glucoside, (-)-epicatechin, and p-coumaroyl hexose (28). Data also demonstrated that all the identified phenolic compounds suffered degradation to some extent during baking (about 84% phenolic compounds' loss calculated by area relationships) or linking to the macromolecules composing biscuit ingredients. These results are in agreement with other bioactive compounds' sources incorporated into biscuit formulations (62). Particularly, procyanidin trimer (Rt = 10.3–10.6 min) presented the highest degradation during baking. By contrast, procyanidin dimer B3, (+)-catechin, and quercetin-3-O-glucoside presented the lowest degradation during baking (50–65% loss of the total area) than the other compounds when compared to the total loss (84%). In this sense, proanthocyanidins have been shown to degrade during heating (100–140.8°C) (63). The smaller loss of procyanidin dimer B3 and (+)-catechin may be explained by their release with the degradation/hydrolysis of procyanidin trimer. The remaining compounds may be responsible for the exhibited antioxidant, α-glucosidase, and pancreatic lipase inhibitory capacities by the biscuits (37). Particularly, phenolic compounds with B rings, such as the ones identified in the TGP biscuit, present inhibition of carbohydrate-hydrolytic enzymes (37). In addition, proanthocyanidins which are mainly present in grape seeds possess many bioactive properties (63).

In brief, the results obtained allow us to hypothesize that the phenolic compounds initially present in TGP could have suffered modifications in their chemical structure by baking temperature or could have ended up forming stronger bonds with the biscuit ingredients during baking that could have impaired the phenolic compounds' extraction during the extraction for analysis (52, 64). Procyanidins and anthocyanins have been shown to degrade at temperatures over 100°C (64). Anthocyanins' chemical structure may have changed because of baking temperature by suffering cleavage, polymerization glycosylation, and/or nucleophilic attack of water (65).

To the best of our knowledge, this is the first study in which the phenolic compounds' loss during the baking of a biscuit with red grape pomace is evidenced by an advanced analytical method such as LC-MS.

## 4. Conclusion

Tannat grape pomace (TGP) microbiological and pesticide analyses showed values within food safety criteria, enabling the use of Uruguayan TGP as a food ingredient. Potential functional biscuits with the nutrition claims of “source of fiber” and “no-added sugar,” varying TGP (10–20% w/w in total wet dough), and sweetener sucralose (2–4% w/w in total wet dough) content were assessed by determining antioxidant, α-glucosidase, and pancreatic lipase inhibition capacities. The highest bioactive properties were exerted by TGP biscuits prepared with the highest substitution of sucralose (20 and 4% w/w total wet biscuit mass, respectively). Moreover, this TGP biscuit presented good sensory quality in a cluster of 37 out of 101 consumers. To the best of our knowledge, this is the first work that the phenolic compounds from TGP and TGP biscuits were identified by HPLC-DAD-MS showing degradation after baking with subsequent changes in phenolic profile. The main phenolic compounds found in TGP biscuit were malvidin-3-O-(6′-p-coumaroyl)glucoside, (+)-catechin, malvidin-3-O-glucoside, and (-)-epicatechin, which may be responsible for the exerted bioactive properties. In conclusion, the results of this study showed the potential of TGP biscuit as a functional food for the risk reduction of chronic diseases and with the concomitant reduction of environmental impact. The remaining phenolic compounds after baking may exert health-promoting properties on the consumers.

The current study is a preliminary study to state the adequate biscuit formulation for the addition of TGP by determining *in vitro* bioactive properties of TGP biscuits without compromising sensory quality. Moreover, this study provided information on the interaction between TGP phenolic compounds and food macro-components by the identification of the free phenolic compounds. In addition, the effect of food processing, especially baking, was also evidenced by the determination of phenolic compounds relative area which is of great importance when thinking about suitable food products for phenolic compounds' efficient health effects. Altogether, the present results show the suitability of TGP as a sustainable functional ingredient in the formulation of biscuits for controlling oxidative stress, glucose, and fatty acid levels.

Nevertheless, antioxidant capacity as well as inhibition of α-glucosidase and pancreatic lipase activities have been reported for changing after digestion (45), thus compromising their effect on human health. Hence, assessment of these properties after simulation of digestion should be addressed to state the remaining bioactive properties and phenolic compounds that may have health-promoting effects. Still, promising potential health benefits are to be expected as many phenolic compounds have been reported for remaining after *in vitro* simulation of digestion or changing into other phenolic compounds that exert bioactive properties, such as proanthocyanidins and their derivatives (63). Moreover, the *in vitro* bioactivities determined in the present work are few of many mechanisms polyphenols may have on human health which are related to metabolic disorders such as diabetes and obesity. In addition, these bioactivities may not be exerted *in vivo*, thus clinical trials should be addressed to confirm the *in vitro* bioactivities determined in the present work.

Beyond determining what happens during digestion, further investigations on the bioactivity of TGP biscuits during the passage of time, as well as microbiological analyses, should be addressed to state their shelf life.

These additional investigations would contribute to a better understanding of the potential applications of grape pomace in food products and its impact on human health.

## Data availability statement

The original contributions presented in the study are included in the article/supplementary material, further inquiries can be directed to the corresponding authors.

## Ethics statement

The studies involving humans were approved by Comité de Ética en Investigación en Seres Humanos de la Facultad de Química, Universidad de la República, General Flores 2124, Montevideo 11800, Uruguay. The studies were conducted in accordance with the local legislation and institutional requirements. The participants provided their written informed consent to participate in this study.

## Author contributions

VO: data curation, formal analysis, investigation, validation, writing—original draft, and writing—review and editing. JB (microbiological analysis): data curation, formal analysis, investigation, and writing—review and editing. RC (HPLC-MS): data curation and investigation. EB (HPLC-MS): data curation. MA (sensory analysis): formal analysis. AG (sensory analysis): data curation, supervision, and writing—review and editing. SA (microbiological analysis): supervision and writing—review and editing. NGG (pesticide analysis): data curation, formal analysis, investigation, methodology, and writing—review and editing. MVC (pesticide analysis): supervision and writing—review and editing. HH (pesticide analysis): supervision and writing—review and editing. ED (HPLC-MS): data curation, methodology, supervision, and writing—review and editing. AMF-F: conceptualization, data curation, formal analysis, funding acquisition, investigation, methodology, project administration, resources, supervision, validation, writing—original draft, and writing—review and editing. AM: conceptualization, funding acquisition, project administration, resources, supervision, validation, and writing—review and editing. All the authors have read and approved the submitted version.
